# Poor Mitochondrial Health and Systemic Inflammation? Testing of a Classical Hypothesis

**DOI:** 10.1093/geroni/igaa057.416

**Published:** 2020-12-16

**Authors:** Marta Zampino, Luigi Ferrucci, Richard Spencer, Kenneth Fishbein, Eleanor Simonsick, Pei-Lun Kuo

**Affiliations:** National Institute on Aging, Bethesda, Maryland, United States

## Abstract

Chronic low-grade inflammation often occurs with aging and has been associated with negative health outcomes. Despite extensive research on the origins of “inflammaging”, the causative mechanisms remain unclear. However, a connection between poor mitochondrial health and chronic inflammation has been hypothesized, with decreasing mitochondrial function occurring with age and precipitating an increase in reactive oxygen species and other pro-inflammatory macromolecules such as mitochondrial DNA. We tested this hypothesis on a population of 619 subjects from the Baltimore Longitudinal Study of Aging, measuring muscle mitochondrial oxidative capacity in vivo by phosphorus magnetic resonance spectroscopy (P-MRS), and plasma interleukin (IL)-6, the most widely used biomarker of inflammaging. The P-MRS-derived post-exercise phosphocreatine recovery time constant tau-PCr, a measure of oxidative capacity, was expressed as a categorical variable through assignment to quintiles. Participants in the first quintile of tau-PCr (best mitochondrial function) were taken as reference and compared to the others using linear regression analysis adjusted for sex, age, lean and fat body mass, and physical activity. Those participants with the lowest oxidative capacity had significantly higher log(IL-6) levels as compared to the reference group. However, data from the other quintiles was not significantly different from the reference values. In conclusion, severe impairment of oxidative capacity is associated with increased inflammation. This study design does not provide conclusive evidence of whether increased inflammation and impaired bioenergetic recovery are both caused by underlying poor health status, or whether mitochondrial deficits lead directly to the observed inflammation; we anticipate addressing this important question with longitudinal studies.

